# Predictors of Multimorbidity (Defined as Diabetes + Hypertension) Amongst Males Aged 15–54 in India: An Urban/Rural Split Analysis

**DOI:** 10.3389/ijph.2024.1606660

**Published:** 2024-02-01

**Authors:** Vikramjit Brar, Ngianga-Bakwin Kandala, Amanda Terry, Amardeep Thind

**Affiliations:** ^1^ Department of Epidemiology and Biostatistics, Western University, London, ON, Canada; ^2^ Interfaculty Program in Public Health, Western University, London, ON, Canada; ^3^ Departments of Family Medicine and Medicine, Schulich School of Medicine and Dentistry, Western University, London, ON, Canada

**Keywords:** diabetes and hypertension, multimorbidity, India, males, predictors of multimorbidity

## Abstract

**Objectives:** This study aimed to determine which sociodemographic and lifestyle factors may act as predictors of multimorbidity (defined as diabetes + hypertension) amongst men aged 15–54 within urban and rural areas of India.

**Methods:** Data from the latest 2019–2021 India NFHS-5 survey were utilized. Presumed cases of multimorbidity were defined as men who had DM + HTN. A total of 22,411 men in urban areas and 66,768 rural men were analyzed using mixed-effect multi-level binary logistic regression models.

**Results:** Various predictors were found to have a statistically significant association to multimorbidity. Urban areas: Age, region of residence, wealth, religion, occupation, and BMI. Rural areas: Age, education, region of residence, wealth, occupation, caste, BMI, alcohol consumption, media exposure, and tobacco consumption.

**Conclusion:** Departing from the broad operational definitions often studied within literature, this study provided insight into one of the most prevalent specific multimorbidities across India. The urban/rural split analyses revealed substantial differences in high-risk characteristics across both areas, which have commonly been overlooked. These findings may better inform policymakers and assist in effectively reducing multimorbidity-related burden through area-specific preventative programs.

## Introduction

As India has continued to experience rapid urbanization and economic development in recent decades, a health and epidemiological transition has followed [1, 2]. The population has been experiencing changing lifestyles, increases in life expectancy, and decreases in overall mortality rates [3, 4]. As such, the prevalence of non-communicable diseases (NCDs) has drastically increased and subsequently an increasing number of individuals have also begun to be diagnosed with multiple NCDs. Due to this, multimorbidity has become an increasing public health concern across India. Multimorbidity is commonly defined as the coexistence of two or more chronic health conditions in an individual [5].

As multimorbidity prevalence continues to increase, its burden is felt not only by the individuals affected but also by the health systems that support them. Affected individuals experience increased levels of disability, treatment burden, financial burden, and overall reduction to quality of life [6, 7]. Multimorbid individuals also often have increased healthcare utilization and require more medical resources [8–10]. Therefore, due to the complex and resource-intensive nature of treating multimorbidity, health systems are burdened. Low- and middle-income countries (LMICs) such as India generally take a vertical approach to healthcare and they are not well equipped to provide the horizontal/patient-focused treatments needed to adequately support multimorbid patients [11, 12].

Diabetes and hypertension are two of the fastest-growing NCDs in India [13, 14], and have been associated with increasing economic burden and NCD-related mortality [15, 16]. Their specific multimorbidity has frequently been reported to be one of the most prevalent with national prevalence estimates ranging from 1.04%–4.7% depending on age demographics of interest [17–19]. More specifically, these conditions have been found to disproportionately affect men, as they have been reported to have increased odds and prevalence of both conditions when compared to women [14, 20].

When studying predictors of multimorbidity, previous studies have generally maintained simple analyses in which residing in urban or rural areas has been treated as another predictor. It has been well established that residing in urban areas increases risk/odds of multimorbidity [8, 21, 22]. However, the literature is lacking in studies that focus on how other predictors may vary across areas due to the varying characteristics of urban and rural residence. As urbanization continues to occur at a rapid pace, urban areas have drastically changed from rural areas, being associated with various positive effects (e.g., improved healthcare, sanitation, improved education, health promotion, etc.) and negative effects (e.g., decreases in physical activity, increased access to processed foods, air pollution, etc.) [23]. Due to these differences across areas, it is of importance to consider how predictors may share differing strengths and directions of association with multimorbidity and that there may also be variations in which predictors are significant in each area. Differing findings for each area have been noted within the minimal literature that has analyzed a split urban and rural analysis [17]. By separating the analysis of predictors into urban and rural areas, high-risk characteristics can be better targeted in each area for more effective preventative and treatment measures [24].

This study aims to fill the gap in the literature regarding the predictors of multimorbidity (defined as diabetes + hypertension) amongst the male population of India, and how predictors vary across urban and rural areas. To obtain the latest population estimates, the recently released National Family Health Survey—5 (NFHS-5) was utilized.

## Methods

### Data Source

For the present study, the source of the data utilized was the NFHS-5, conducted between 2019 and 2021. The Ministry of Health and Family Welfare (MoHFW), Government of India, was responsible for both funding and conducting each NFHS with support from the International Institute for Population Sciences (IIPS), Mumbai, and ICF, United States [25]. The ICF contributed technical assistance through the Demographic and Health Survey (DHS) program.

The NFHS-5 followed a stratified two-stage sampling method. All 707 districts recognized in India at the time were considered for sampling with each district being stratified into urban and rural areas. For the first stage of sampling, urban and rural samples were obtained. To obtain rural samples, villages in rural areas were selected as primary sampling units (PSUs) utilizing a probability proportional to size (PPS) method and similarly for urban areas, Census Enumeration Blocks (CEBs) were selected as PSUs [25]. Each PSU was treated as a rural or urban cluster and from these clusters, the second stage of sampling was conducted in which 22 random households were selected from each cluster using an equal probability systematic selection [25]. Collected data pertained to 101,839 men aged 15–54 and 724,115 women aged 15–49 across 636,399 households [24]. These data included demographics, socioeconomic characteristics, health-related data, and various anthropometric/biomarker data (height, weight, blood pressure, blood glucose, etc.). To obtain data relevant to the men sampled, the NFHS-5 “Male Recode” and “household recode” datasets were merged [26]. All 101,839 men sampled were eligible to be included in our analyses (with exclusions arising from subsequent missing data/modeling constraints).

### Variables

#### Outcome/Dependent Variable

The outcome of interest was multimorbidity, which we defined as the co-existence of both diabetes and hypertension within an individual. As clinical confirmation was not available, we aimed to determine any possible cases, and thus, broad criteria were established for defining presumed cases of diabetes and hypertension.

Criteria for diabetes and hypertension diagnosis were set as follows:

(a) Diabetes: If a respondent replied yes to or met at least one of the following criteria they were categorized as a possible case of diabetes: 1) “Do you currently have diabetes?” 2) “Are you currently taking prescribed medication to lower your blood glucose level?” 3) “Have you been told you have high blood glucose on 2 or more occasions by a doctor or other health professional?” 4) A non-fasting plasma glucose level reading of ≥ 200 mg/dL. 5) A fasting plasma glucose level reading of ≥ 126 mg/dL (Respondents were defined as being in a fasting state if they self-reported having not eaten or drank anything (except water) for 8 continuous hours prior to their glucometer blood test [27]).
(b) Hypertension: If a respondent replied yes to or met at least one of the following criteria they were categorized as a possible case of hypertension: 1) “Do you currently have hypertension?” 2) “Are you currently taking prescribed medication to lower your blood pressure?” 3) “Have you been told you have high blood pressure on 2 or more occasions by a doctor or other health professional?” 4) Average systolic blood pressure reading of ≥ 135 mmHg and an average diastolic blood pressure reading of ≥ 85 mmHg ( ≥ 135/85). Indian context cut-off for hypertension diagnosis using any automated oscillometric device as per the Indian Guidelines of Hypertension–Edition 4 [28]).

A distribution of responses/readings for each of these variables can be seen within Supplementary File S1.

#### Independent Variables

Based on an extensive literature review conducted regarding predictors of multimorbidity across India [8, 13, 17, 21, 22, 29–32], a variety of individual-level characteristics were considered for this study which could best be grouped as 1) sociodemographic factors and 2) lifestyle factors. Sociodemographic factors included: age, education, region of residence, wealth, religion, current marital status, occupation, and caste. Lifestyle factors included: Body mass index (BMI), alcohol consumption, consumption of a healthy diet, media exposure, and tobacco consumption.

Age was treated as a continuous variable ranging from 15 years to 54 years old. Education was categorized into: “no education,” “incomplete primary”—(1–5 years of education), “incomplete secondary” (6–11 years of education), and “completed secondary or higher”—(12+ years of education). Region of residence was categorized into geographic areas of India: “North,” “South,” “East,” “West,” “Central,” and “North-East.” Wealth was defined using the wealth index produced by the DHS Program. Within this wealth index, respondents were categorized into quintiles of increasing wealth [33]. Categories of wealth were as follows, “poorest” (first quintile), “poorer” (second quintile), “middle” (third quintile), “richer” (fourth quintile), and “richest” (fifth quintile). Religion was categorized into “Hindu,” “Muslim,” “Christian,” and “other”—(Sikh, Buddhist/Neo-Buddhist, Jain, Jewish, Parsi/Zoroastrian, other or no religion). Current marital status was categorized as “married” or “single” with the latter being defined as those who self-reported being divorced, widowed, deserted, never been married, or are no longer living together/separated from their partner. Occupations were categorized into: “not working,” “agricultural,” “professional/services/technical/managerial/clerical/sales,” “skilled and unskilled manual,” and “other.” Caste was categorized into “scheduled caste” (SC), “scheduled tribe” (ST), “other backward class” (OBC), and “none.”

BMI was categorized based on values ranges relevant to the Indian population context [34]: “Underweight”—(BMI<18.5 $\left.\;kg/m^{2}\right)$ , “Normal”—( $\left.18.5\;kg/m^{2}\leq BMI<23\;kg/m^{2}\right)$ , “overweight”—( $\left.23\;kg/m^{2}\leq BMI<25\;kg/m^{2}\right)$ , and “obese”—( $25\;kg/m^{2}\leq BMI$ ). Media exposure was a constructed variable that took into consideration each respondent’s use of radio, television, newspaper/magazine, mobile phones, and internet. For each respondent, a discrete score was computed ranging from 0–5 by summing up the number of media sources they self-reported as using. Alcohol consumption was treated as a binary variable, with those who self-reported any consumption being coded as “yes.” Similarly, tobacco consumption was treated as a binary variable with any respondent who self-reported consumption of cigarettes, bidis, or tobacco in any other form such as cigar, pipe, hookah, gutkha/paan masala with tobacco, khaini, pan with tobacco, other chewing tobacco, snuff, etc., being categorized as “yes.” Lastly, consumption of a healthy diet was also treated as a binary variable with those coded as “yes” being respondents who reported daily or weekly consumption of both fruits and dark leafy green vegetables.

### Statistical Analysis

STATA 17, edition BE (Basic), was used to conduct all relevant tasks such as merging datasets, defining/creating variables, and conducting analyses. For univariate analysis, first descriptive statistics were explored. For the continuous/discrete variables (age and media exposure), mean, standard deviation, median, and interquartile range (IQR) values were determined. For all other variables (categorical/binary), frequency distribution of observations was reported. To test if differences in univariate findings across urban and rural areas were statistically significant, chi-square tests ( $\chi^{2}$ ) and Mann-Whitney tests were conducted respectively for categorical/binary and continuous/discrete variables. To avoid type 1 error inflation for these multiple comparisons, the Bonferroni correction was applied.

For bivariate analysis, an unadjusted binary logistic regression was run for each independent variable with multimorbidity to determine unadjusted odds ratios (OR). For multivariable analysis, multi-level mixed-effect binary logistic regression models were employed. Two separate multivariable models were conducted to determine adjusted associations between predictors of interest and multimorbidity separately for urban area and rural area respondents. Both models took into consideration all sociodemographic and lifestyle factors previously discussed. Furthermore, both models accounted for districts as the cluster variable (random intercept) to adjust for any possible clustering effect. To test the goodness-of-fit of each multivariable model, likelihood ratio tests (LRT) were conducted against a regular non-multilevel binary logistic regression [35]. All analyses were conducted using unweighted data due to the unavailability of NFHS-5 sampling weights.

## Results

Table 1 shows the univariate characteristics. For all variables analyzed within the univariate analysis, differences across urban and rural areas were found to be statistically significant (*p* < 0.001) (as per Chi-square and Mann-Whitney tests). Denoting these statistically significant findings is an asterisk (*) beside each variable within Table 1.

**TABLE 1:** Univariate analysis—Multimorbidity and sample characteristics split by urban and rural areas, India, 2020–2021.

Variable	Urban	Rural
	*n* (%)	*n* (%)
Dependent Variable		
Multimorbidity^a^ (*n* = 94,243)		
No	23,013 (96.2%)	68,733 (97.7%)
Yes	909 (3.8%)	1,588 (2.3%)
Independent Variables		
Sociodemographic factors		
Age^a^ (years)		
Mean ± SD	32.5 ± 11.1	32.1 ± 11.3
Median (IQR)	32.0 (23.0; 41.0)	31.0 (22.0; 41.0)
Education^a^		
No education	1,875 (7.1%)	10,394 (13.8%)
Incomplete primary	2,268 (8.6%)	9,442 (12.5%)
Incomplete secondary	14,046 (53.2%)	41,761 (55.4%)
Completed secondary or higher	8,231 (31.1%)	13,822 (18.3%)
Region^a^		
North India	6,368 (24.1%)	14,766 (19.6%)
Central India	4,692 (17.8%)	18,550 (24.6%)
East India	3,057 (11.5%)	12,140 (16.1%)
North-East India	2,979 (11.3%)	11,881 (15.7%)
West India	3,873 (14.7%)	7,715 (10.2%)
South India	5,451 (20.6%)	10,367 (13.8%)
Wealth Index^a^		
Poorest (1st Quintile)	786 (3.0%)	19,010 (25.2%)
Poorer (2nd Quintile)	2,238 (8.5%)	20,361 (27.0%)
Middle (3rd Quintile)	4,580 (17.3%)	17,135 (22.7%)
Richer (4th Quintile)	7,921 (30.0%)	12,288 (16.3%)
Richest (5th Quintile)	10,895 (41.2%)	6,625 (8.8%)
Religion^a^		
Hindu	19,301 (73.0%)	57,910 (76.8%)
Muslim	4,297 (16.3%)	7,815 (10.4%)
Christian	1,661 (6.3%)	5,606 (7.4%)
Other	1,161 (4.4%)	4,088 (5.4%)
Current Marital Status^a^		
Single (never in union, divorcee, widowed, separated, deserted)	10,747 (40.7%)	27,715 (36.8%)
Married	15,673 (59.3%)	47,704 (63.2%)
Occupation^a^		
Professional/services/Technical/managerial/clerical/sales	9,954 (37.8%)	11,574 (15.4%)
Not working	5,359 (20.3%)	13,882 (18.5%)
Agricultural	1,929 (7.3%)	30,935 (41.1%)
Skilled and unskilled manual	7,723 (29.3%)	16,207 (21.5%)
Other	1,394 (5.3%)	2,639 (3.5%)
Caste^a^		
Scheduled caste	4,719 (19.0%)	14,541 (20.2%)
Scheduled tribe	2,694 (10.8%)	16,660 (23.1%)
OBC (other backward classes)	10,820 (43.5%)	28,506 (39.6%)
None	6,649 (26.7%)	12,319 (17.1%)
lifestyle factors		
BMI^a^		
Underweight	2,738 (11.2%)	11,144 (15.6%)
Normal	9,192 (37.8%)	33,671 (47.1%)
Overweight	4,844 (19.9%)	12,605 (17.6%)
Obese	7,568 (31.1%)	14,146 (19.7%)
Alcohol Consumption^a^		
No	20,066 (75.9%)	55,325 (73.4%)
Yes	6,354 (24.1%)	20,094 (26.6%)
Healthy Diet^a^		
No	9,913 (37.5%)	40,133 (53.2%)
Yes	16,507 (62.5%)	35,286 (46.8%)
Media exposure^a^ (score 0–5)		
Mean ± SD	3.4 ± 1.2	2.8 ± 1.3
Median (IQR)	3.0 (3.0; 4.0)	3.0 (2.0; 4.0)
Tobacco consumption^a^		
No	16,941 (64.1%)	40,671 (53.9%)
Yes	9,479 (35.9%)	34,748 (46.1%)

Note: 1) Unweighted analysis.

^a^ Statistically significant Chi-square test and Mann-Whitney test results between urban and rural findings denoted with asterisks besides independent variable. (Bonferroni correction applied). *p < 0.001, **p < 0.01, ***p < 0.05.

Within urban areas, a statistically significant difference was found in multimorbidity prevalence with 3.8% of men being defined as multimorbid in urban areas, and a lesser 2.3% in rural areas. Ages ranged from 15–54 in both areas, however the difference in mean age of respondents across areas was found to be statistically significant. In urban areas the mean (32.5 ± 11.1 years) and median (32.0 years) ages were slightly greater as compared to rural areas, where mean age was 32.1 ± 11.3 years and median age was 31.0 years.

In urban areas a greater proportion of men were found to have higher levels of education. Furthermore, a clear disparity in wealth was found as the majority of men in urban areas belonged to the richer quintiles of wealth, but the opposite was true in rural areas, with the majority belonging to the poorer quintiles. Religious and marital status composition is similar across both areas with a majority of men being Hindu (∼75%) and married (∼60%). Within rural areas, the most common occupation was agriculture (41.1%) and in urban areas the most predominate category of occupations was professional, services, technical, managerial, clerical, and sales-related (37.8%). Caste composition was similar across both areas.

In terms of lifestyle characteristics, statistically significant differences existed across areas. A greater proportion of men in urban areas having higher BMIs when compared to those in rural areas (Table 1). Within urban areas a greater proportion (62.5%) of men consumed “healthy diets” as compared to rural areas (46.8%). Men in urban areas also had greater media exposure with an average score of 3.4 as compared to 2.8 in rural areas. Finally, alcohol consumption was similar across both areas but urban areas had a greater proportion of men self-reporting tobacco consumption (46.1%) as compared to rural areas (35.9%).

Table 2 describes both bivariate and multivariable associations between predictors of interest and multimorbidity within urban areas. Multivariable analysis found that age, region of residence, wealth, religion, occupation, and BMI each had statistically significant associations to multimorbidity. Each year, increase in age was associated with an 10% increase in odds of multimorbidity [AOR = 1.10; (95% CI: 1.09, 1.12)]. When compared to those residing in Northern India, men residing in the Eastern, Southern, and Western regions respectively had 1.65 (95% CI: 1.14, 2.38), 1.50 (95% CI: 1.08, 2.06), and 0.64 (95% CI: 0.43, 0.96) times the odds of multimorbidity. Wealth had a positive association to multimorbidity, with men belonging to the “richer” (fourth) and “richest” (fifth) quintiles of wealth respectively having 2.14 (95% CI: 1.09, 4.17) and 2.34 (95% CI: 1.18, 4.62) times the odds of multimorbidity as compared to those from the “poorest” quintile (first). Christians were found to have 38% reduced odds of multimorbidity [AOR = 0.62; (95% CI: 0.40, 0.95)] when compared to Hindus. Men working skilled/unskilled manual labor jobs had 22% reduced odds of multimorbidity [AOR = 0.78; (95% CI: 0.65, 0.95)], when compared to those working professional, services, technical, managerial, clerical, and sales jobs. Overweight and obese men respectively had 1.40 (95% CI: 1.12, 1.75) and 2.09 (95% CI: 1.72, 2.54) times the odds of multimorbidity as compared to men with normal BMI. Furthermore, the intraclass correlation coefficient (ICC) was reported to be 0.15 in urban areas meaning approximately 15% of the variability in multimorbidity outcome can be attributed to district of residence. The LRT test yielded a value of 98.46 (*p* < 0.001), meaning the chosen multi-level model is a better fit for the analysis.

**TABLE 2:** Bivariate and multivariable analyses of sociodemographic and lifestyle predictors of multimorbidity for urban area residents, India, 2020–2021.

Independent variables	Unadjusted OR (95% CI)	*p*	Adjusted OR (95% CI)	*p*
Sociodemographic factors				
Age	**1.11 (1.10, 1.12)**	<0.001	**1.10 (1.09, 1.12)**	<0.001
Education				
No education	**—**		**—**	
Incomplete primary	1.14 (0.83, 1.57)	0.409	1.12 (0.79, 1.59)	0.518
Incomplete secondary	0.88 (0.68, 1.14)	0.336	1.00 (0.73, 1.37)	0.990
Completed secondary or higher	0.93 (0.71, 1.22)	0.603	1.00 (0.71, 1.42)	0.991
Region				
North India	**—**		**—**	
Central India	0.90 (0.72, 1.12)	0.344	1.17 (0.83, 1.63)	0.370
East India	**1.30 (1.04, 1.64)**	0.023	**1.65 (1.14, 2.38)**	0.008
North-East India	1.25 (0.99, 1.58)	0.057	1.46 (0.96, 2.22)	0.073
West India	**0.57 (0.43, 0.75)**	<0.001	**0.64 (0.43, 0.96)**	0.030
South India	**1.55 (1.28, 1.87)**	<0.001	**1.50 (1.08, 2.06)**	0.014
Wealth Index				
Poorest (1st Quintile)	**—**		**—**	
Poorer (2nd Quintile)	1.30 (0.66, 2.54)	0.449	1.22 (0.60, 2.49)	0.581
Middle (3rd Quintile)	**2.05 (1.10, 3.80)**	0.024	1.81 (0.92, 3.53)	0.084
Richer (4th Quintile)	**2.55 (1.39, 4.69)**	0.002	**2.14 (1.09, 4.17)**	0.026
Richest (5th Quintile)	**3.10 (1.69, 5.66)**	<0.001	**2.34 (1.18, 4.62)**	0.015
Religion				
Hindu	**—**		**—**	
Muslim	**0.62 (0.50, 0.77)**	<0.001	0.76 (0.58, 1.00)	0.053
Christian	0.83 (0.62, 1.10)	0.194	**0.62 (0.40, 0.95)**	0.027
Other	0.88 (0.63, 1.22)	0.442	0.95 (0.63, 1.43)	0.814
Current Marital Status				
Single	**—**		**—**	
Married	**6.54 (5.26, 8.15)**	<0.001	1.32 (1.00, 1.75)	0.053
Occupation				
Professional/services/Technical/managerial/clerical/sales	**—**		**—**	
Not working	**0.25 (0.19, 0.32)**	<0.001	1.31 (0.95, 1.82)	0.098
Agricultural	0.80 (0.62, 1.02)	0.074	0.84 (0.63, 1.11)	0.222
Skilled and unskilled manual	**0.64 (0.55, 0.76)**	<0.001	**0.78 (0.65, 0.95)**	0.011
Other	0.87 (0.66, 1.15)	0.322	1.02 (0.74, 1.40)	0.920
Caste				
None	**—**		**—**	
Scheduled caste	**0.72 (0.58, 0.89)**	0.003	0.93 (0.72, 1.19)	0.558
Scheduled tribe	1.03 (0.81, 1.30)	0.818	1.34 (0.95, 1.88)	0.097
OBC (other backward classes)	1.03 (0.87, 1.21)	0.744	1.07 (0.87, 1.30)	0.525
Lifestyle factors				
BMI				
Normal	**—**		**—**	
Underweight	**0.40 (0.26, 0.63)**	<0.001	0.65 (0.40, 1.06)	0.084
Overweight	**2.08 (1.70, 2.56)**	<0.001	**1.40 (1.12, 1.75)**	0.004
Obese	**3.60 (3.03 4.29)**	<0.001	**2.09 (1.72, 2.54)**	<0.001
Alcohol Consumption				
No	**—**		**—**	
Yes	**1.57 (1.36, 1.81)**	<0.001	1.15 (0.97, 1.37)	0.118
Healthy Diet				
No	**—**		**—**	
Yes	**1.27 (1.11, 1.47)**	0.001	1.06 (0.90, 1.25)	0.485
Media exposure (score 0–5)	**1.10 (1.04, 1.17)**	0.001	1.02 (0.94, 1.10)	0.689
Tobacco consumption				
No	**—**		**—**	
Yes	**1.24 (1.08, 1.42)**	0.002	1.12 (0.94, 1.33)	0.218
Random effect (Random Intercept)	Variance of intercepts (95% CI)			
Group Variable: Districts	0.57 (0.41, 0.79)			
Intraclass correlation coefficient (95% CI)	LR test—multivariable analysis			
0.15 (0.11, 0.19)	Multi-level model vs. regular binary logistic regression: = 98.46 (*p* < 0.001)			

Note: 1) Bivariate and multivariable both unweighted analyses 2) Bolded odds ratio text indicates statistically significant findings.

Table 3 similarly describes bivariate and multivariable findings but, for rural areas. Multivariable analysis found that age, education, region of residence, wealth, occupation, caste, BMI, alcohol, media exposure, and tobacco each had statistically significant associations with multimorbidity. Each year increase in age was associated with an 9% increase in odds of multimorbidity [AOR: 1.09 (95% CI: 1.09, 1.10)]. When compared to those residing in Northern India, men residing in the Central, Eastern, Norther-Eastern, and Southern regions respectively had 1.41 (95% CI: 1.11, 1.79), 2.20 (95% CI: 1.72, 2.83), 1.58 (95% CI: 1.19, 2.10), and 1.87 (95% CI: 1.47, 2.38) times the odds of multimorbidity. Wealth had a positive association to multimorbidity with each subsequent increasing quintile of wealth being associated with increase odds of multimorbidity. Men working skilled/unskilled manual labor and agricultural jobs respectively had 18% [AOR = 0.78; (95% CI: 0.65, 0.95)] and 29% [AOR = 0.71; (95% CI: 0.61, 0.82)] reduced odds of multimorbidity, when compared to those working professional, services, technical, managerial, clerical, and sales jobs. Men in SCs had 1.20 (95% CI: 1.00, 1.44) times the odds of multimorbidity as compared to those in none of the highly marginalized castes.

**TABLE 3:** Bivariate and multivariable analyses of sociodemographic and lifestyle predictors of multimorbidity for rural area residents, India, 2020–2021.

Independent variables	Unadjusted OR (95% CI)	*p*	Adjusted OR (95% CI)	*p*
Sociodemographic factors				
Age	**1.09 (1.09, 1.10)**	<0.001	**1.09 (1.09, 1.10)**	<0.001
Education				
No education	**—**		**—**	
Incomplete primary	1.16 (0.96, 1.39)	0.123	**1.30 (1.06, 1.59)**	0.010
Incomplete secondary	0.93 (0.80, 1.07)	0.315	**1.24 (1.04, 1.49)**	0.019
Completed secondary or higher	0.87 (0.73, 1.05)	0.141	1.13 (0.89, 1.43)	0.304
Region				
North India	**—**		**—**	
Central India	0.98 (0.83, 1.16)	0.842	**1.41 (1.11, 1.79)**	0.005
East India	**1.51 (1.27, 1.78)**	<0.001	**2.20 (1.72, 2.83)**	<0.001
North-East India	1.19 (1.00, 1.42)	0.053	**1.58 (1.19, 2.10)**	0.002
West India	**0.79 (0.63, 0.99)**	0.045	0.94 (0.69, 1.27)	0.680
South India	**2.07 (1.76, 2.44)**	<0.001	**1.87 (1.47, 2.38)**	<0.001
Wealth Index				
Poorest (1st Quintile)	**—**		**—**	
Poorer (2nd Quintile)	**1.30 (1.10, 1.52)**	0.002	**1.20 (1.00, 1.44)**	0.046
Middle (3rd Quintile)	**1.72 (1.47, 2.01)**	<0.001	**1.42 (1.17, 1.72)**	<0.001
Richer (4th Quintile)	**2.08 (1.77, 2.45)**	<0.001	**1.53 (1.24, 1.90)**	<0.001
Richest (5th Quintile)	**2.60 (2.17, 3.11)**	<0.001	**1.93 (1.50, 2.47)**	<0.001
Religion				
Hindu	**—**		**—**	
Muslim	1.02 (0.87, 1.20)	0.805	1.22 (0.98, 1.52)	0.076
Christian	0.89 (0.73, 1.09)	0.273	0.82 (0.62, 1.09)	0.170
Other	1.20 (0.97, 1.47)	0.086	1.01 (0.78, 1.32)	0.924
Current Marital Status				
Single	**—**		**—**	
Married	**4.42 (3.79, 5.15)**	<0.001	1.03 (0.84, 1.24)	0.802
Occupation				
Professional/services/Technical/managerial/clerical/sales	**—**		**—**	
Not working	**0.20 (0.16, 0.25)**	<0.001	0.87 (0.67, 1.13)	0.286
Agricultural	**0.59 (0.52, 0.67)**	<0.001	**0.71 (0.61, 0.82)**	<0.001
Skilled and unskilled manual	**0.59 (0.51, 0.69)**	<0.001	**0.82 (0.69, 0.96)**	0.016
Other	**0.74 (0.57, 0.96)**	0.026	1.01 (0.76, 1.33)	0.969
Caste				
None	**—**		**—**	
Scheduled caste	0.92 (0.78, 1.08)	0.317	**1.20 (1.00, 1.44)**	0.048
Scheduled tribe	**0.70 (0.59, 0.82)**	<0.001	0.97 (0.78, 1.20)	0.770
OBC (other backward classes)	0.92 (0.80, 1.06)	0.247	1.02 (0.87, 1.19)	0.818
Lifestyle factors				
BMI				
Normal	—		—	
Underweight	**0.52 (0.41, 0.65)**	<0.001	**0.77 (0.60, 0.98)**	0.032
Overweight	**1.96 (1.70, 2.25)**	<0.001	**1.46 (1.26, 1.70)**	<0.001
Obese	**3.63 (3.22, 4.09)**	<0.001	**2.28 (2.00, 2.60)**	<0.001
Alcohol Consumption				
No	—		—	
Yes	**1.55 (1.40, 1.73)**	<0.001	**1.25 (1.10, 1.42)**	<0.001
Healthy Diet				
No	—		—	
Yes	**1.17 (1.06, 1.29)**	0.002	1.00 (0.89, 1.12)	0.999
Media exposure (score 0–5)	**1.11 (1.07, 1.15)**	<0.001	**1.08 (1.02, 1.14)**	0.005
Tobacco consumption				
No	—		—	
Yes	**1.11 (1.00, 1.23)**	0.036	**0.86 (0.76, 0.97)**	0.017
Random effect (Random Intercept)	Variance of intercepts (95% CI)			
Group Variable: Districts	0.31 (0.24, 0.41)			
Intraclass correlation coefficient (95% CI)	LR test—multivariable analysis			
0.09 (0.07, 0.11)	Multi-level model vs. regular binary logistic regression: = 122.73 (*p* < 0.001)			

Note: 1) Bivariate and multivariable both unweighted analyses 2) Bolded odds ratio text indicates statistically significant findings.

Underweight, overweight, and obese men respectively had 0.77 (95% CI: 0.60, 0.98), 1.46 (95% CI: 1.26, 1.70) and 2.28 (95% CI: 2.00, 2.60) times the odds of multimorbidity as compared to men with normal BMI. Men who consumed alcohol had 25% increased odds of multimorbidity [AOR = 1.25; (95% CI: 1.10, 1.42)] as compared to those who self-reported consumption of no alcohol. For each unit increase in media exposure score, odds of multimorbidity increased by 8% [AOR = 1.08; (95% CI: 1.02, 1.14)]. Lastly, tobacco consumption was instead found to be protective in rural areas with an AOR of 0.86 (95% CI: 0.76, 0.97).

The intraclass correlation coefficient (ICC) was reported to be 0.09 in rural areas meaning approximately 9% of the variability in multimorbidity outcome can be attributed to district of residence. The LRT test yielded a value of 122.73 (*p* < 0.001), meaning the chosen multi-level model is the better fit for the analysis.

## Discussion

### Key Findings

Within this study, various population characteristics and their associations to multimorbidity amongst men aged 15–54 was explored. Prevalence of multimorbidity was found to be greater in urban areas as compared to rural. Additionally urban and rural areas were found to have statistically significant differences in regard to sociodemographic and lifestyle characteristics. However, as previously mentioned, sampling weights were unavailable for the NFHS-5. As such, data could not be weighted prior to analysis making all univariate findings unweighted. With existing literature commonly presenting national prevalence estimates based on weighted data, our univariate findings are incomparable.

### Interpretation and Implications

With various discrepancies being found across urban and rural areas regarding predictors, it becomes of interest how the causal relationship between predictors and multimorbidity is affected by area. It is possible that area specific characteristics act as effect modifiers and alter the found effect of predictors across the strata of urban and rural areas. As such, the urban/rural perspective becomes ever important for public health interest. When further interpreting the significant findings of this study and difference across urban and rural areas, we interpret the findings as follows.

Consistent with literature, age was found to have a positive association to multimorbidity [8, 13, 21, 29–32]. As individuals age, it is thought that susceptibility to the accumulation of chronic health conditions is accelerated due to the progressive dysregulation of organ systems [36]. Similarly, positive association findings regarding the wealth-multimorbidity association were also consistent with the literature. However, it is of interest that in rural areas, all quintiles were statistically significant, but in urban areas, this was true only for the two richest (fourth and fifth) quintiles. It is possible that due to urban areas having improved health systems, the progression of NCDs is better controlled, and that it is not until much greater levels of wealth are considered that negative implications of wealth become unmanageable [37]. Previous studies have reported, for the occupation-multimorbidity association, that physically intensive jobs reduce odds of multimorbidity [12]. This study expands on such findings as specific physically intensive occupations were found to have protective effects in both urban and rural areas. Regions of residence had no clear trend in findings, but variation in odds across regions can likely be associated to highly diverse characteristics such as nutrition, rate of urbanization, environment, healthcare, etc. Furthermore, increasing BMI, increasing media exposure, and consumption of alcohol each resulted in increased odds of multimorbidity, thus having findings consistent with the literature [8, 21, 29–32]. However, the latter two predictors were solely significant in rural areas. Surprising was the finding that tobacco consumption had a protective effect in rural areas. Such findings are not expected as tobacco has commonly been accepted as a risk factor for many health conditions. However, social desirability bias must be considered due to the self-reported nature of this variable. If respondents mispresented their risky lifestyle behaviors, such erroneous findings may be explained. Another predictor found to have an unclear association with multimorbidity was education. However, education has frequently been reported to have a varying association with multimorbidity based on operational definition [38].

The findings of this study have implications for not only men across India but also relevant policymakers who act to protect the health of the population. Currently, across India, health policies such as preventative plans have been quite basic. One such example has been the use of the World Health Organization (WHO) “*Action Plan of Global Strategy for the Prevention and Control of Noncommunicable Diseases*” to control the progression of NCDs, and therefore subsequently multimorbidity. Within this action plan, the WHO highlights basic guidelines that may assist in improving population health [39, 40]. India, being a WHO member state, has committed to incorporating the objectives and goals of this plan into various national public health programs [39]. Some basic examples from this plan are the focus on reducing alcohol consumption, improving diets, and increased physical activity [40]. However, since this action plan was made for many nations ranging from high-income to LMICs, it fails to acknowledge that common associations between risk factors and health outcomes may not be consistent across all nations. In LMICs such as India, the drastic differences across urban and rural areas may reduce the efficacy of national health programs, with more areas specific programs being required. One such example from this study is that while certain factors such as BMI may be a statistically significant predictor of multimorbidity across India, alcohol is only significant within rural areas. Policymakers can benefit from such findings as they can be better informed on risk factors in each area allowing for more efficient and effective resource allocation. By focusing efforts such as screening and health promotion amongst those who possess high-risk characteristics in each area, there may be earlier diagnosis of multimorbidity, possibly resulting in reduced complications and associated burdens.

Furthermore, the implications on the health systems of India must be considered. Currently, there exists a mixed framework for healthcare in which there are both public and private (for-profit and not-for-private) providers [41]. Funding many of the public facilities is publicly funded health insurance (PFHI) [42]. However, compared to other nations, India has minimal allocation to their healthcare when considered as a percentage of their GDP [43]. As such, public services have often been associated with reduced quality and inadequacies in resources [41]. Many individuals have opted for private care which is usually an out-of-pocket expense (OOPE) [41]. However, with rural areas being associated with lesser wealth (when compared to urban), this creates further disparity in access to healthcare. As a result of these OOPE inequities, universal health coverage (UHC) has become a goal for India with programs such as the “*Ayushman Bharat*” program and “*Pradhan Mantri Jan Arogya Yojana*” being established [42]. These programs aim to create more health wellness centers and provide additional coverage to those families that may need it [42]. The findings of this study can be used to inform relevant parties regarding the extensive list of predictors of multimorbidity across both areas. With a greater number of sociodemographic and lifestyle factors being found to be associated to multimorbidity in rural areas (as compared to urban), this may incentivize governments to allocate further funding to rural areas where OOPEs may not be feasible for most residents. This would allow policymakers to create programs that better support those who otherwise may receive little to no support. Additionally, as we determined which population characteristics have increased odds of multimorbidity across both areas, new UHC programs such as those previously discussed can use such information to better design screening and intervention protocols.

### Limitations

This study is not without its limitations, specifically with the data and methodology. Firstly, due to the cross-sectional nature of the NFHS data analyzed, causality between the dependent (multimorbidity) and independent variables cannot be established. Furthermore, since the majority of the data collected in the NFHS was self-reported, it is possible that respondents may introduce response bias if they answer questions inaccurately, regardless of whether or not it is intentional. One specific example may be social desirability bias, in which respondents may provide inaccurate information regarding sensitive topics such as tobacco consumption, alcohol consumption, or caste.

Due to the previously mentioned lack of availability of sampling weights, all analyses in this study were unweighted. Therefore, the univariate findings of this study could not be compared to the literature. Lastly, the limitation in the multimorbidity outcome variable must be acknowledged. To define multimorbidity, various data elements were utilized from the NFHS-5. However, under such a definition, any case of multimorbidity found is presumed multimorbidity. Found cases are not clinically confirmed cases that are firm in diagnosis but rather diagnoses based on the information we had available.

### Conclusion

Being one of the first studies to focus on diabetes and hypertension multimorbidity in India, this study provides an in-depth understanding of one of the most prevalent specific multimorbidities. This study identified high-risk characteristics in both urban and rural areas through its split analysis and contributes a better understanding of predictors to the literature. Furthermore, this was done using the latest data available, making findings reflective of the current male population of India. This study sets a precedent for further focused research into specific multimorbidities. Findings can be used by relevant policymakers and health systems across India so they can better support those affected by multimorbidity. As India continues to face public health issues, such studies may be crucial in controlling the associated burdens.

## Data Availability

All data utilized within this study is publicly available on the Demographic and Health Surveys (DHS) official website. Upon creating an account, datasets can be requested for use at the following link (https://dhsprogram.com).

## References

[1] Basto-Abreu A Barrientos-Gutierrez T Wade AN Oliveira de Melo D Semeão de Souza AS Nunes BP Multimorbidity Matters in Low and Middle-Income Countries. J Multimorbidity Comorbidity (2022) 12:26335565221106074. 10.1177/26335565221106074 PMC9208045 35734547

[2] Luna F Luyckx VA. Why Have Non-Communicable Diseases Been Left Behind? Asian Bioeth Rev (2020) 12(1):5–25. 10.1007/s41649-020-00112-8 33717328 PMC7747415

[3] Nethan S Sinha D Mehrotra R. Non Communicable Disease Risk Factors and Their Trends in India. Asian Pac J Cancer Prev (2017) 18(7):2005–10. 10.22034/APJCP.2017.18.7.2005 28749643 PMC5648412

[4] Yadav S Arokiasamy P. Understanding Epidemiological Transition in India. Glob Health Action (2014) 7(1):23248. 10.3402/gha.v7.23248 24848651 PMC4028906

[5] WHO. Multimorbidity: Technical Series on Safer Primary Care. Geneva: World Health Organization (2016). Licence: CC BY-NC-SA 3.0 IGO.

[6] Rosbach M Andersen JS. Patient-Experienced Burden of Treatment in Patients With Multimorbidity – A Systematic Review of Qualitative Data. PLOS ONE (2017) 12(6):e0179916. 10.1371/journal.pone.0179916 28644877 PMC5482482

[7] Laires PA Perelman J. The Current and Projected Burden of Multimorbidity: A Cross-Sectional Study in a Southern Europe Population. Eur J Ageing (2018) 16(2):181–92. 10.1007/s10433-018-0485-0 31139032 PMC6509308

[8] Khan MR Malik MA Akhtar SN Yadav S Patel R. Multimorbidity and its Associated Risk Factors Among Older Adults in India. BMC Public Health (2022) 22(1):746. 10.1186/s12889-022-13181-1 35422020 PMC9008964

[9] Soley-Bori M Ashworth M Bisquera A Dodhia H Lynch R Wang Y Impact of Multimorbidity on Healthcare Costs and Utilisation: A Systematic Review of the UK Literature. Br J Gen Pract (2020) 71(702):e39–e46. 10.3399/bjgp20x713897 33257463 PMC7716874

[10] Pati S Agrawal S Swain S Lee JT Vellakkal S Hussain MA Non Communicable Disease Multimorbidity and Associated Health Care Utilization and Expenditures in India: Cross-Sectional Study. BMC Health Serv Res (2014) 14(1):451. 10.1186/1472-6963-14-451 25274447 PMC4283077

[11] Prathapan S Fernando GV Matthias AT Bentota Mallawa Arachchige Charuni Y Abeygunawardhana HM Somathilake BG. The Rising Complexity and Burden of Multimorbidity in a Middle-Income Country. PLOS ONE (2020) 15(12):e0243614. 10.1371/journal.pone.0243614 33306724 PMC7732070

[12] Balakrishnan S Karmacharya I Ghimire S Mistry SK Singh DR Yadav OP Prevalence of Multimorbidity and its Correlates Among Older Adults in Eastern Nepal. BMC Geriatr (2022) 22(1):425. 10.1186/s12877-022-03115-2 35570271 PMC9109315

[13] Puri P Singh SK Pati S. Temporal Dynamics, Patterns and Correlates of Single and Multimorbidity in India, 1994–2018. J Multimorbidity Comorbidity (2021) 11:26335565211062756. 10.1177/26335565211062756 PMC8728765 35004339

[14] Anjana RM Unnikrishnan R Deepa M Pradeepa R Tandon N Das AK Metabolic Non-Communicable Disease Health Report of India: The ICMR-INDIAB National Cross-Sectional Study (ICMR-INDIAB-17). Lancet Diabetes Endocrinol (2023) 11(7):474–89. 10.1016/s2213-8587(23)00119-5 37301218

[15] Sahoo SK Pathni AK Krishna A Sharma B Cazabon D Moran AE Financial Implications of Protocol-Based Hypertension Treatment: An Insight Into Medication Costs in Public and Private Health Sectors in India. J Hum Hypertens (2022) 37:828–34. 10.1038/s41371-022-00766-x 36271130 PMC10471490

[16] Das H Moran AE Pathni AK Sharma B Kunwar A Deo S. Cost-Effectiveness of Improved Hypertension Management in India Through Increased Treatment Coverage and Adherence: A Mathematical Modeling Study. Glob Heart (2021) 16(1):37. 10.5334/gh.952 34040950 PMC8121007

[17] Prenissl J De Neve J-W Sudharsanan N Manne-Goehler J Mohan V Awasthi A Patterns of Multimorbidity in India: A Nationally Representative Cross-Sectional Study of Individuals Aged 15 to 49 Years. PLOS Glob Public Health (2022) 2(8):e0000587. 10.1371/journal.pgph.0000587 36962723 PMC10021201

[18] Sinha A Kerketta S Ghosal S Kanungo S Lee JT Pati S. Multimorbidity and Complex Multimorbidity in India: Findings From the 2017–2018 Longitudinal Ageing Study in India (LASI). Int J Environ Res Public Health (2022) 19(15):9091. 10.3390/ijerph19159091 35897461 PMC9332385

[19] Mini GK Thankappan KR. Pattern, Correlates and Implications of Non-Communicable Disease Multimorbidity Among Older Adults in Selected Indian States: A Cross-Sectional Study. BMJ Open (2017) 7(3):e013529. 10.1136/bmjopen-2016-013529 PMC5353268 28274966

[20] Neupane D McLachlan CS Sharma R Gyawali B Khanal V Mishra SR Prevalence of Hypertension in Member Countries of South Asian Association for Regional Cooperation (SAARC): Systematic Review and Meta-Analysis. Medicine (2014) 93(13):e74. 10.1097/md.0000000000000074 25233326 PMC4616265

[21] Puri P Singh SK. Patterns and Predictors of Non-Communicable Disease Multimorbidity Among Older Adults in India: Evidence From Longitudinal Ageing Study in India (LASI), 2017–2018. J Public Health Pol (2022) 43(1):109–28. 10.1057/s41271-021-00321-x 34997210

[22] Chauhan S Patel R Kumar S. Prevalence, Factors and Inequalities in Chronic Disease Multimorbidity Among Older Adults in India: Analysis of Cross-Sectional Data From the Nationally Representative Longitudinal Aging Study in India (LASI). BMJ Open (2022) 12(3):e053953. 10.1136/bmjopen-2021-053953 PMC8961109 35351706

[23] Ranzani OT Kalra A Di Girolamo C Curto A Valerio F Halonen JI Urban-Rural Differences in Hypertension Prevalence in Low-Income and Middle-Income Countries, 1990–2020: A Systematic Review and Meta-Analysis. PLOS Med (2022) 19(8):e1004079. 10.1371/journal.pmed.1004079 36007101 PMC9410549

[24] Janes H Pepe MS Gu W. Assessing the Value of Risk Predictions by Using Risk Stratification Tables. Ann Intern Med (2008) 149(10):751–60. 10.7326/0003-4819-149-10-200811180-00009 19017593 PMC3091826

[25] International Institute for Population Sciences (IIPS) and ICF. National Family Health Survey (NFHS-5), 2019-21. India. Mumbai: IIPS (2021).

[26] International Institute for Population Sciences (IIPS) and ICF. National Family Health Survey (NFHS-5) 2019-21 [Dataset]. IAMR7DFL.Dta & ]. IAHR7DFL.Dta: Ministry of Health and Family Welfare (MoHFW), Government of India. ICF, USA: USAID [Producers] ICF [distributor] (2021).

[27] American Diabetes Association. Diagnosis of Diabetes. Diagnosis | ADA (2022). Available at: https://diabetes.org/diabetes/a1c/diagnosis (Accessed December 29, 2022).

[28] Shah SN Munjal YP Kamath SA Wander GS Mehta N Mukherjee S Indian Guidelines on Hypertension-IV (2019). J Hum Hypertens (2020) 34(11):745–58. 10.1038/s41371-020-0349-x 32427886

[29] Singh K Patel SA Biswas S Shivashankar R Kondal D Ajay VS Multimorbidity in South Asian Adults: Prevalence, Risk Factors and Mortality. J Public Health (2018) 41(1):80–9. 10.1093/pubmed/fdy017 PMC7304513 29425313

[30] Mishra VK Srivastava S Murthy PV. Population Attributable Risk for Multimorbidity Among Adult Women in India: Do Smoking Tobacco, Chewing Tobacco and Consuming Alcohol Make a Difference? PLOS ONE (2021) 16(11):e0259578. 10.1371/journal.pone.0259578 34731220 PMC8565748

[31] Debsarma D Saha J Choudhary BK. Prevalence, Pattern, and Correlates of Multimorbidity Among Adult and Old Aged Women in India. Clin Epidemiol Glob Health (2022) 17:101143. 10.1016/j.cegh.2022.101143

[32] Puri P Kothavale A Singh SK Pati S. Burden and Determinants of Multimorbidity Among Women in Reproductive Age Group: A Cross-Sectional Study Based in India. Wellcome Open Res (2021) 5:275. 10.12688/wellcomeopenres.16398.2 34131591 PMC8182697

[33] Rutstein SO Johnson. K. The DHS Wealth Index. Calverton, Maryland: ORC Macro (2004). DHS Comparative Reports No. 6.

[34] World Health Organization (WHO). Regional Office for the Western Pacific. (2000).The Asia-Pacific Perspective: Redefining Obesity and its Treatment. Sydney: Health Communications Australia. Available at: https://apps.who.int/iris/handle/10665/206936 (Accessed June 16, 2023).

[35] Gelman A Hill J. Data Analysis Using Regression and Multilevel/Hierarchical Models. Cambridge: Cambridge University Press (2021).

[36] Fabbri E Zoli M Gonzalez-Freire M Salive ME Studenski SA Ferrucci L. Aging and Multimorbidity: New Tasks, Priorities, and Frontiers for Integrated Gerontological and Clinical Research. J Am Med Directors Assoc (2015) 16(8):640–7. 10.1016/j.jamda.2015.03.013 PMC5125299 25958334

[37] Banerjee S. Determinants of Rural-Urban Differential in Healthcare Utilization Among the Elderly Population in India. BMC Public Health (2021) 21(1):939. 10.1186/s12889-021-10773-1 34001026 PMC8130530

[38] Feng X Kelly M Sarma H. The Association Between Educational Level and Multimorbidity Among Adults in Southeast Asia: A Systematic Review. PLOS ONE (2021) 16(12):e0261584. 10.1371/journal.pone.0261584 34929020 PMC8687566

[39] Nethan S Sinha D Mehrotra R. Non Communicable Disease Risk Factors and Their Trends in India. Asian Pac J Cancer Prev : APJCPvol. (2017) 18:2005–10. 10.22034/APJCP.2017.18.7.2005 PMC5648412 28749643

[40] WHO. Global Action Plan for the Prevention and Control of Noncommunicable Diseases 2013-2020. Geneva, Switzerland: World Health Organization – WHO document Production Services (2013). ISBN: 978 92 4 150623 6.

[41] Selvaraj S Karan KA Srivastava S Bhan N Mukhopadhyay I. India Health System Review. New Delhi: World Health Organization, Regional Office for South-East Asia (2022).

[42] Reshmi B Unnikrishnan B Rajwar E Parsekar SS Vijayamma R Venkatesh BT. Impact of Public-Funded Health Insurances in India on Health Care Utilisation and Financial Risk Protection: A Systematic Review. BMJ open (2021) 11:e050077. 10.1136/bmjopen-2021-050077 PMC8704974 34937714

[43] Narain JP. Public Health Challenges in India: Seizing the Opportunities. Indian J Community Med (2016) 41(2):85–8. 10.4103/0970-0218.177507 27051080 PMC4799645

