# Supplementary material for: Predictors of Multimorbidity (Defined as Diabetes + Hypertension) Amongst Males Aged 15–54 in India: An Urban/Rural Split Analysis
Source: Int J Public Health. 2024 Feb 1;69:1606660. doi: 10.3389/ijph.2024.1606660 (PMC10867130; doi:10.3389/ijph.2024.1606660)

**Supplementary File 1:**

This supplemental document contains graphical distributions of each variable considered in this studies outcome variable. First presented is the distribution of all variables considered when defining a presumed case of diabetes. Following is the distribution of all variables considered when defining a presumed case of hypertension.

The criteria for diabetes and hypertension diagnosis were set as follows:

1. Diabetes: If a respondent replied yes to/met at least one of the following criteria they were categorized as a possible case of diabetes: 1) “Do you currently have diabetes?” 2) “Are you currently taking prescribed medication to lower your blood glucose level?” 3) “Have you been told you have high blood glucose on 2 or more occasions by a doctor or other health professional?” 4) A non-fasting plasma glucose level reading of $\geq$ 200 mg/dL. 5) A fasting plasma glucose level reading of $\geq$ 126 mg/dL.
2. Hypertension: If a respondent replied yes to/met at least one of the following criteria they were categorized as a possible case of hypertension: 1) “Do you currently have hypertension?” 2) “Are you currently taking prescribed medication to lower your blood pressure?” 3) “Have you been told you have high blood pressure on 2 or more occasions by a doctor or other health professional?” 4) Average systolic blood pressure reading of $\geq$ 135mmHg and an average diastolic blood pressure reading of $\geq$ 85mmHg ($\geq$ 135/85).

**Diabetes:**

**(6.13%) of respondents were presumed to be diabetic based on the following variables.**

Figure 1: “Do you currently have diabetes?”


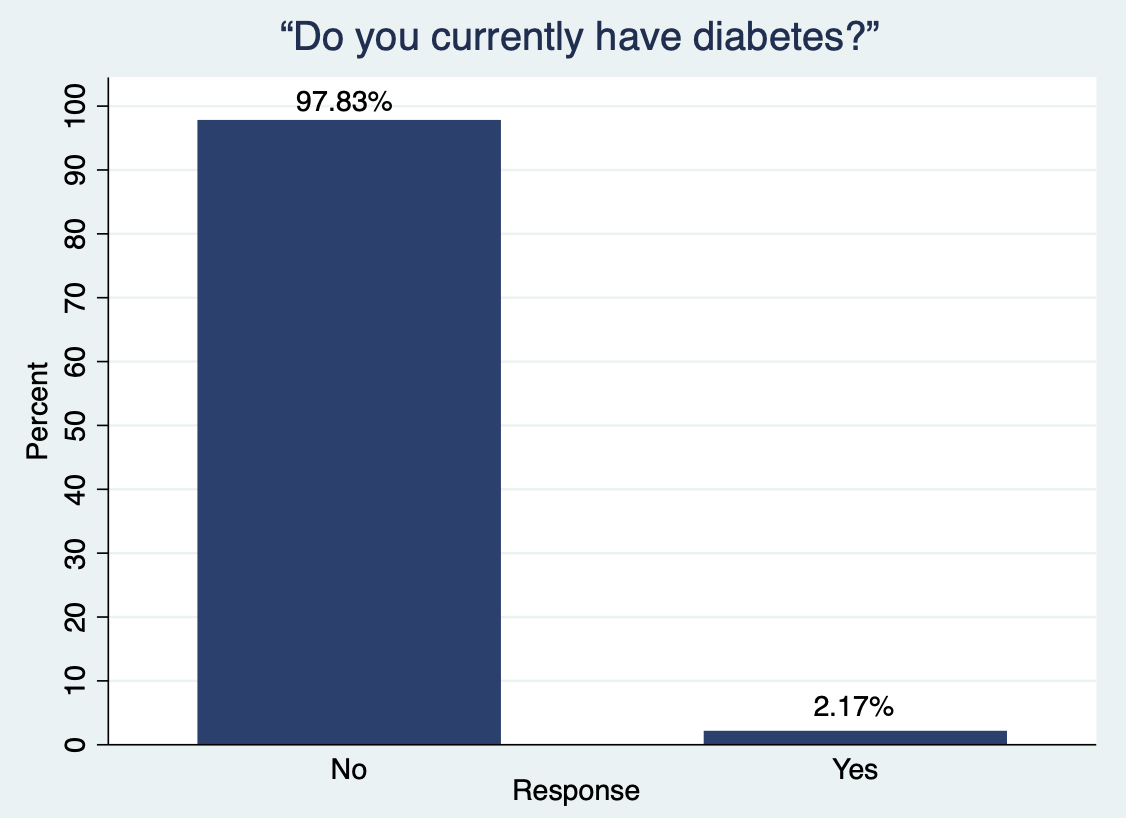


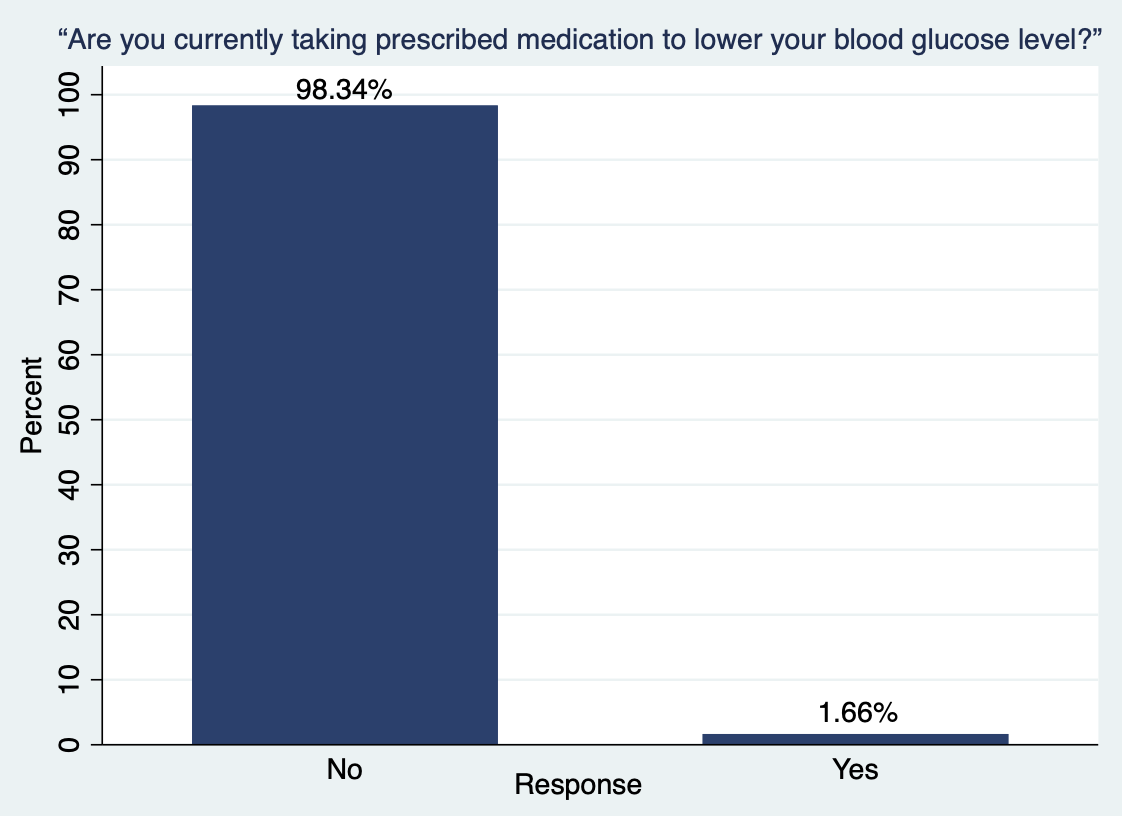
Figure 2: “Are you currently taking prescribed medication to lower your blood glucose level?”


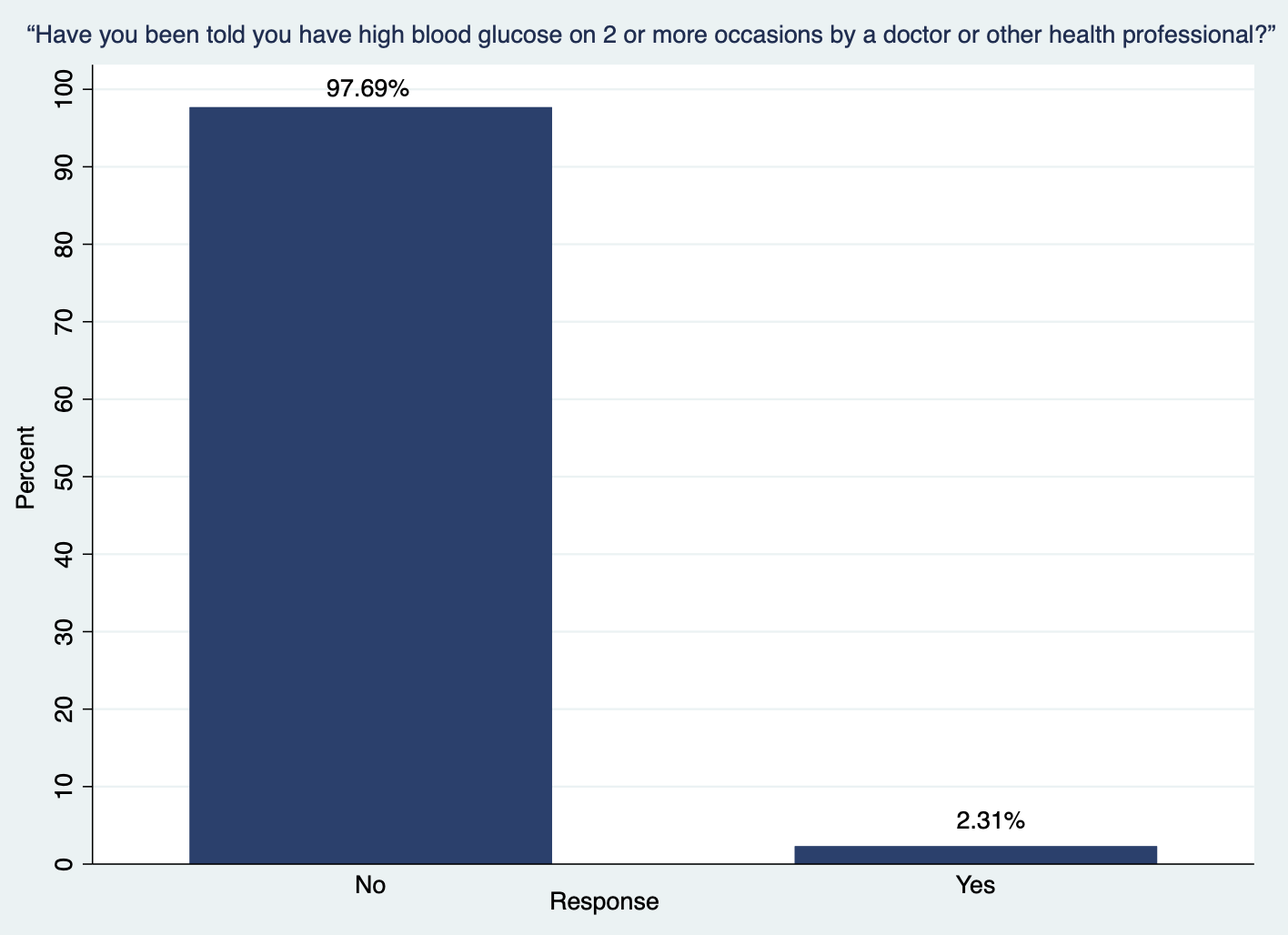
Figure 3: “Have you been told you have high blood glucose on 2 or more occasions by a doctor or other health professional?”

Figure 4: A non-fasting plasma glucose level reading of $\geq$ 200 mg/dL.


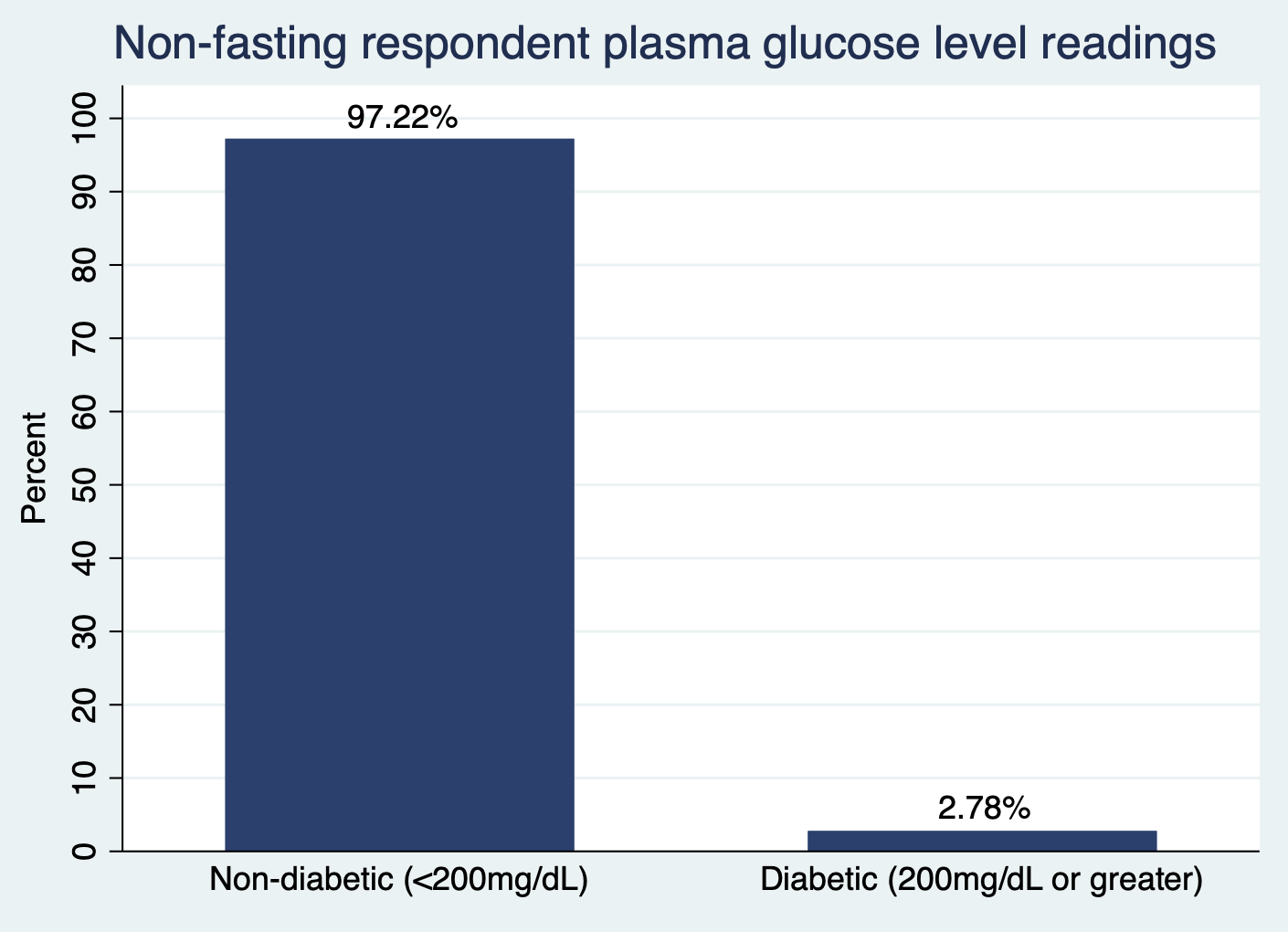


Figure 5: A fasting plasma glucose level reading of $\geq$ 126 mg/dL.


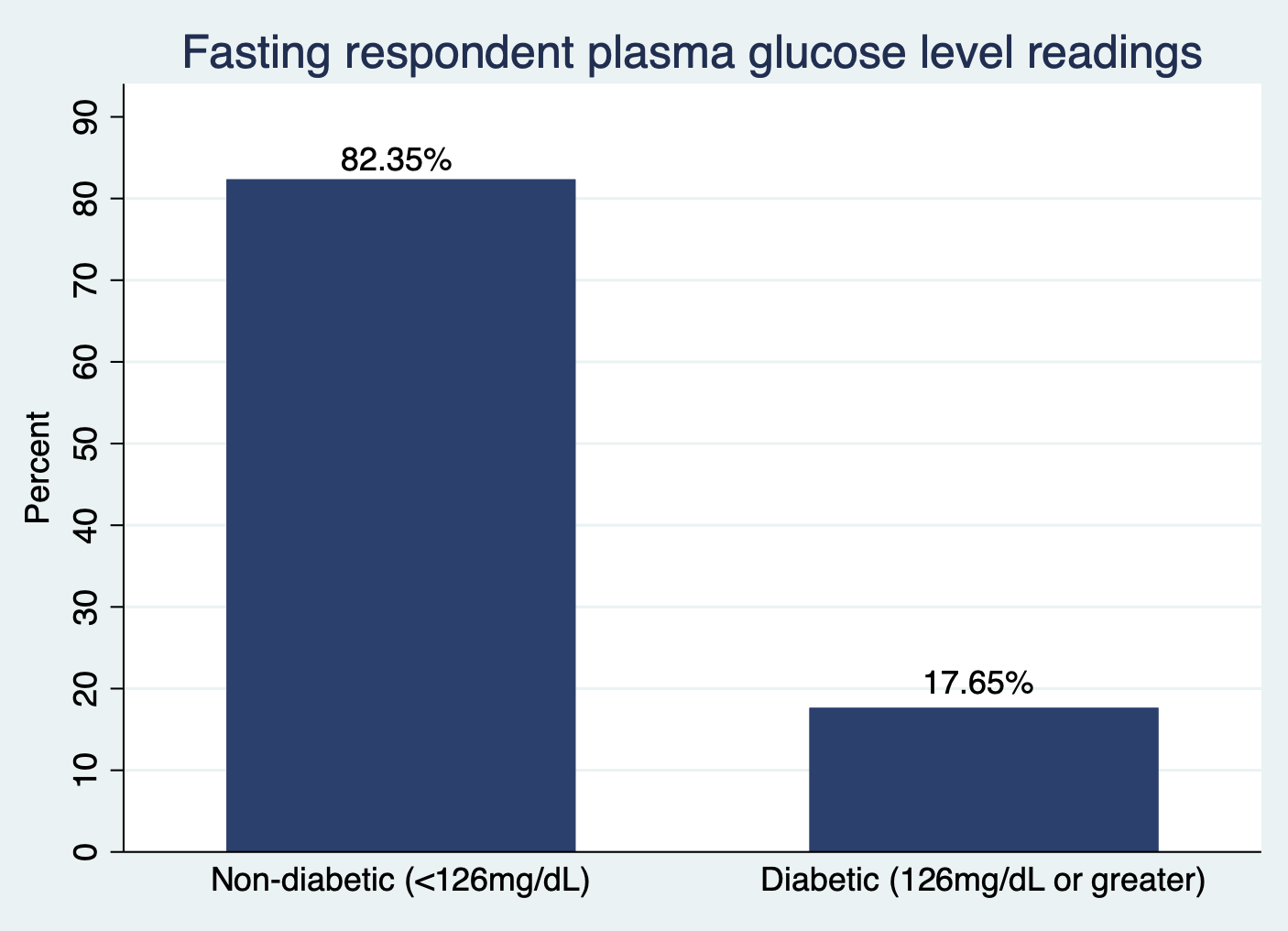


**Hypertension:**

**(17.73%) of respondents were presumed to be hypertensive based on the following variables.**

Figure 6: “Do you currently have hypertension?”


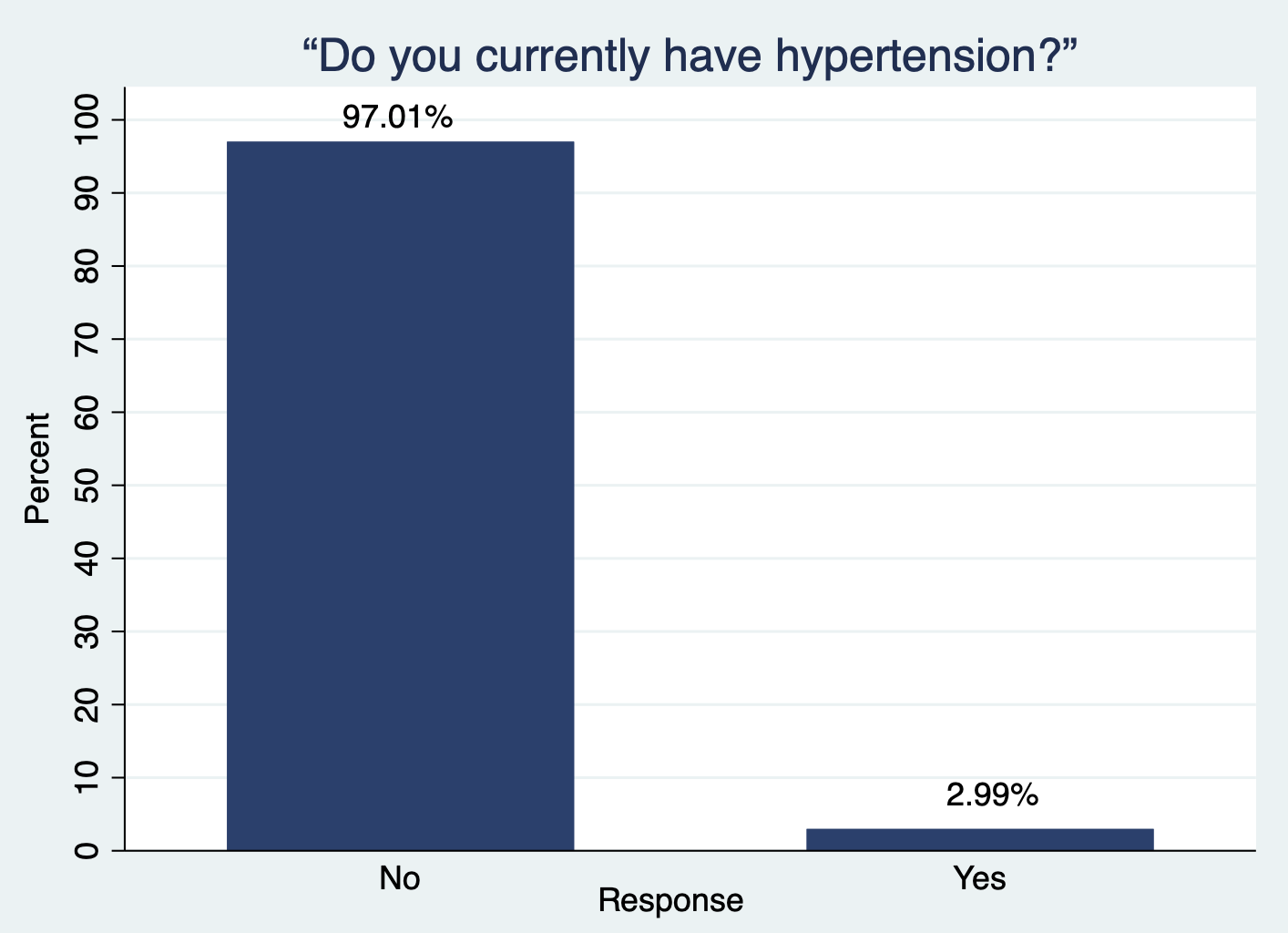


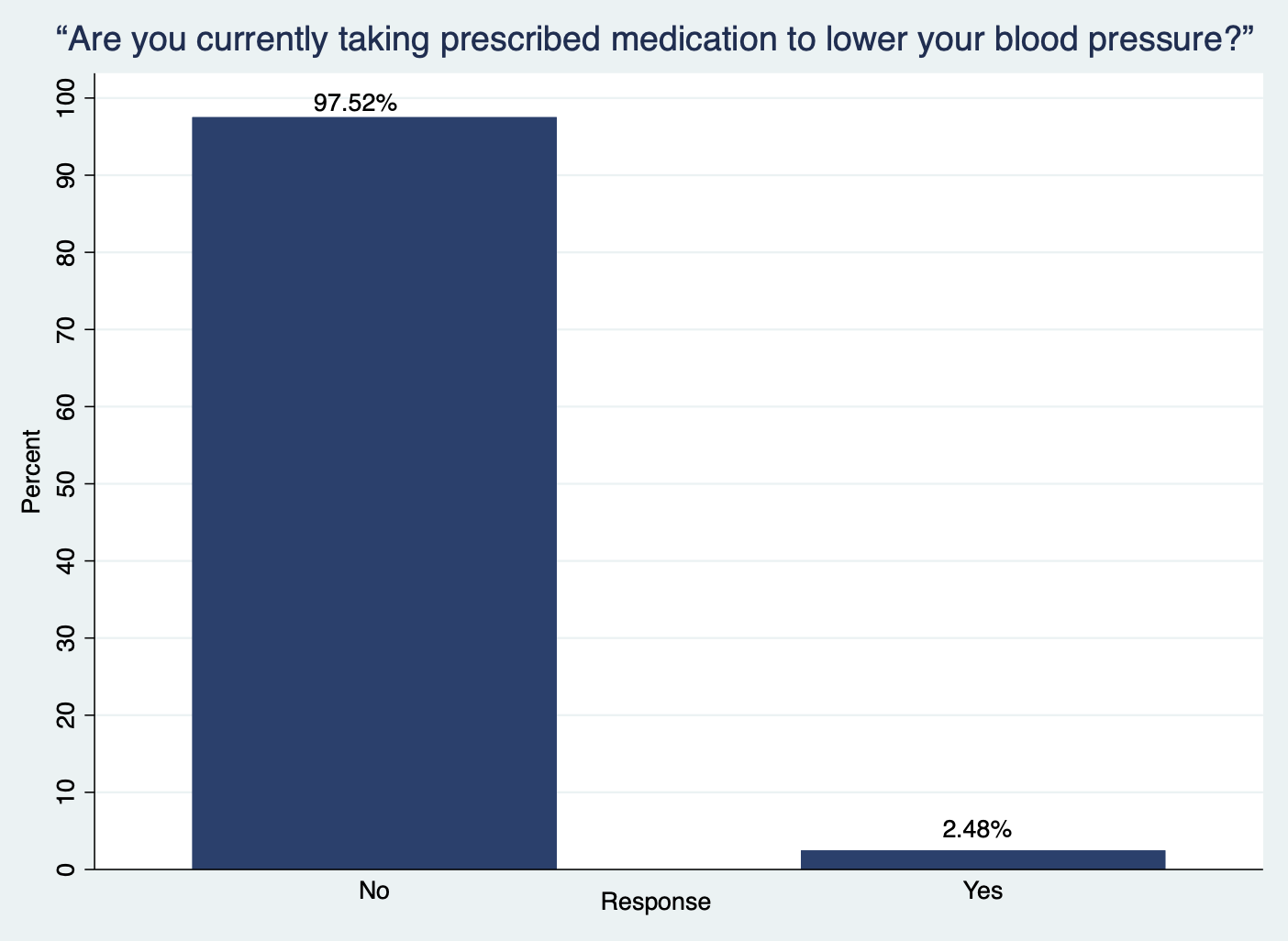
Figure 7: “Are you currently taking prescribed medication to lower your blood pressure?”

Figure 8: “Have you been told you have high blood pressure on 2 or more occasions by a doctor or other health professional?”


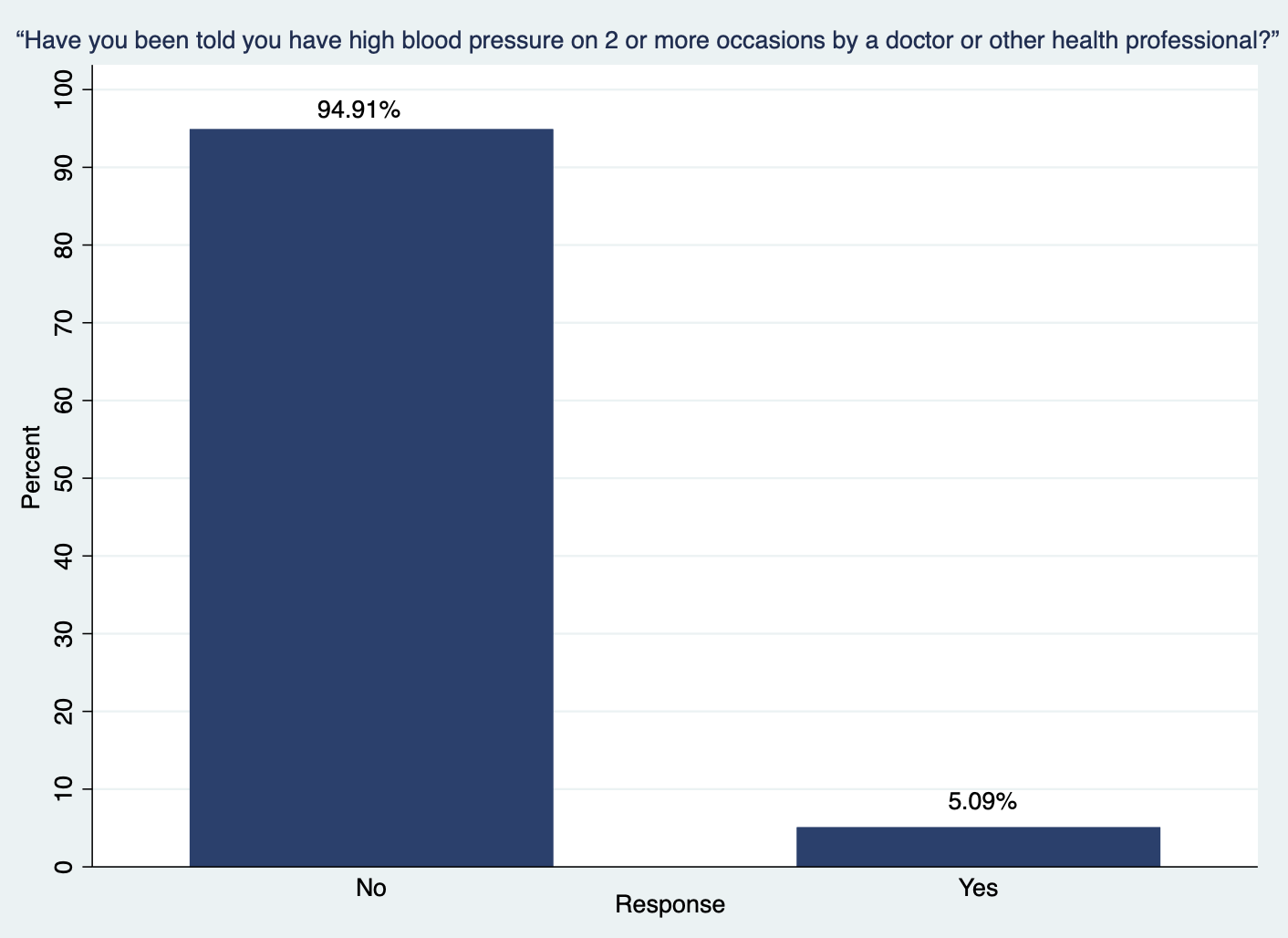


Figure 9: Average systolic blood pressure reading of $\geq$ 135mmHg and an average diastolic blood pressure reading of $\geq$ 85mmHg ($\geq$ 135/85).


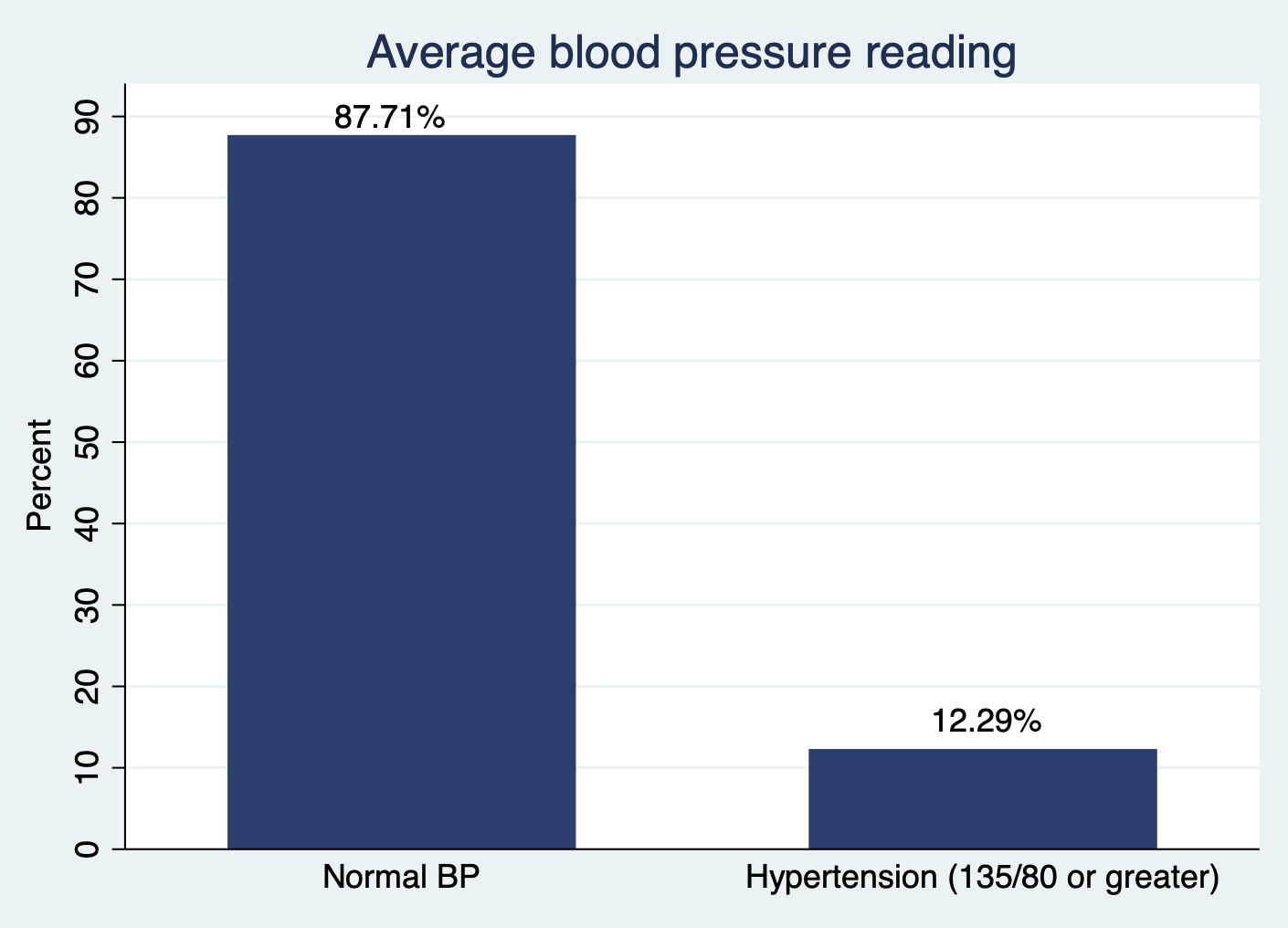

Supplement: Supplementary file 1 [file DataSheet1.docx]
